# Chronic TGFβ stimulation promotes the metastatic potential of lung cancer cells by Snail protein stabilization through integrin β3-Akt-GSK3β signaling

**DOI:** 10.18632/oncotarget.8295

**Published:** 2016-03-23

**Authors:** Gab-Yong Bae, Soon-Ki Hong, Jeong-Rak Park, Ok-Seon Kwon, Keun-Tae Kim, JaeHyung Koo, Ensel Oh, Hyuk-Jin Cha

**Affiliations:** ^1^ College of Natural Sciences, Department of Life Sciences, Sogang University, Seoul, Republic of Korea; ^2^ Department of Brain and Cognitive Sciences, DGIST, Daegu, Republic of Korea; ^3^ Laboratory of Cancer Genomics and Molecular Pathology, Samsung Biomedical Research Institute, Samsung Medical Center, Seoul, Republic of Korea

**Keywords:** chronic TGFβ exposure, integrin β3, Akt, GSK3β, Snail

## Abstract

Chronic exposure to TGFβ, a frequent occurrence for tumor cells in the tumor microenvironment, confers more aggressive phenotypes on cancer cells by promoting their invasion and migration while at the same time increasing their resistance to the growth-inhibitory effect of TGFβ. In this study, a transdifferentiated (TD) A549 cell model, established by chronically exposing A549 cells to TGFβ, showed highly invasive phenotypes in conjunction with attenuation of Smad-dependent signaling. We show that Snail protein, the mRNA expression of which strongly correlates with a poor prognosis in lung cancer patients, was highly stable in TD cells after TGFβ stimulation. The increased protein stability of Snail in TD cells correlated with elevated inhibitory phosphorylation of GSK3β, resulting from the high Akt activity. Notably, integrin β3, whose expression was markedly increased upon sustained exposure to TGFβ, was responsible for the high Akt activity as well as the increased Snail protein stability in TD cells. Consistently, clinical database analysis on lung cancer patients revealed a negative correlation between overall survival and integrin β3 mRNA levels. Therefore, we suggest that the integrin β3-Akt-GSK3β signaling axis plays an important role in non-canonical TGFβ signaling, determining the invasive properties of tumor cells chronically exposed to TGFβ.

## INTRODUCTION

TGFβ signaling plays an important role in tumor suppression as well as tumor promotion events such as initiation, progression and metastasis [1]. As a tumor promoter, TGFβ, the prototype of the TGFβ superfamily, confers metastatic potential to tumor cells by inducing epithelial-mesenchymal transition (EMT), characterized by the acquisition of invasive phenotypes. Conversely, growth-inhibitory or apoptosis-inducing effects of TGFβ suppress tumor progression [2]. The dual roles of TGFβ, as either a tumor promoter or a tumor suppressor, depend on the stage of tumor development; it serves as a tumor promoter for aggressive tumors and a tumor suppressor for normal cells or early carcinoma [3]. However, the molecular mechanisms underlying these different responses are still unclear despite extensive studies and numerous hypotheses [1, 4, 5].

Considering that tumor microenvironments are often enriched in secreted TGFβ [6], cancer cells are considered to be chronically exposed to TGFβ. Consistently, emerging reports demonstrate that after chronic TGFβ exposure, cancer cells acquire resistance to the growth-inhibitory effect of TGFβ and concurrently acquire malignancy or/and invasive potential (e.g., EMT). Adaptation to chronic TGFβ exposure is achieved via alterations in the signaling networks of tumor cells that make them more prone to malignant progression [7–9].

Resistance to TGFβ-mediated growth arrest is attributed to loss-of-function mutations or reduced expression of TGFβ signaling components [10], including Smad4, the common mediator of Smad-dependent (canonical) TGFβ signaling [11]. Paradoxically, attenuation of TGFβ signaling can also reduce tumor cell malignancy by causing the defective production of key EMT-regulatory factors such as *ZEB1*, *TWIST*, *SLUG* and *SNAIL* [12]. Therefore, in this regard, cancer malignancy can be seen as a corollary of the abrogation of the tumor suppressive effect of TGFβ and the simultaneous potentiation of its tumor-promoting effect, mediated by alterations in TGFβ signaling pathways.

TGFβ receptor activation by TGFβ also transmits signals to Smad-independent (non-canonical) pathways through the mitogen activated protein kinase (MAPK) pathway, Rho-like GTPase pathway, and phosphatidylinositol-3-kinase (PI3K)/Akt pathway [13]. Interestingly, the protein stability of key EMT-regulatory factors, such as Twist, Slug and Snail, is also regulated by ERK, Akt, and GSK3β [14–16]. Thus, it is highly plausible that the acquisition of dependence on non-canonical TGFβ pathways accounts for the promotion of cancer malignancy even in the absence of *SMAD4* [17, 18].

In the present study, we established transdifferentiated (TD) cells by exposing A549 tumor cells to chronic TGFβ exposure [7], which revealed skewed signaling toward Akt-GSKβ. The increased invasive properties of TD cells were associated with Akt/GSK3β-mediated up-regulation of Snail protein. We also reasoned that integrin β3 might direct TGFβ signals toward Akt and GSK3β to stabilize Snail. Consistently, clinical database analysis revealed a poor survival outcome for cancer patients with high integrin β3 mRNA expression, suggesting that the integrin β3-Akt-GSK3β signaling axis could be used as an important therapeutic target for preventing malignant cancer progression.

## RESULTS

### Chronic TGFβ stimulation enhances tumor cell migration and invasion

**Figure 1:**
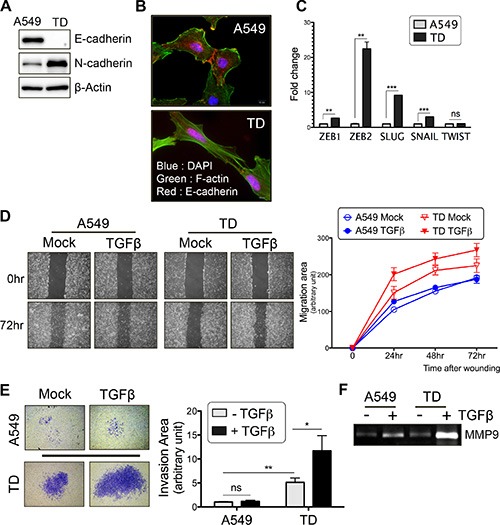
Chronic TGFβ stimulation enhances tumor cell migration and invasion (**A**) Immunoblotting with E-cadherin or N-cadherin antibody. β-actin was used as a loading control. (**B**) The cell-cell adhesion was visualized by immunostaining with E-cadherin antibody (Red) and cytoskeletal organization was visualized by F-actin staining with Phalloidin (Green). DAPI (Blue) was used to counterstain nuclear. (**C**) Quantitative real-time PCR for measuring the mRNA levels of EMT-genes in basal culture condition. (**D**) Wound healing assay was performed after TGFβ treatment for 24 hours. Representative images (left) and migration rate (right graph) are shown. (**E**) Two chamber-matrigel invasion assay was performed after TGFβ treatment for 24 hours. Invading cells across the membranes (left images) and invasion capacity (right graph) are shown. (**F**) After TGFβ treatment for 24 hours, cell culture supernatants were subjected to zymography to measure MMP9 activity. *Columns*, means ± SE. *P < 0.05; **P < 0.01; ***P < 0.001; ns, non-significant.

In this study, chronic exposure of A549 tumor cells to TGFβ was sufficient by itself to induce EMT, which is characterized by reduced E-cadherin expression and increased N-cadherin expression and cytoskeletal reorganization (see F-actin staining in green; Figure 1A and 1B). Additionally, real-time PCR analysis revealed that typical EMT markers, such as *ZEB1, ZEB2, SLUG* and *SNAIL*, were highly expressed in TD cells (Figure 1C). Consistent with these EMT characteristics, TD cells showed greater migration and invasion than control A549 cells. Moreover, additional TGFβ stimulation further promoted the cell migration (Figure 1D) and invasion (Figure 1E) of TD cells, but not that of control A549 cells. The increased invasiveness of TD cells after chronic exposure to TGFβ occurred concomitantly with the up-regulation of MMP9 activity (Figure 1F). TD cells also exhibited resistance to TGFβ-induced growth inhibition, whereas control cells exhibited sensitivity to the inhibitory effects of TGFβ on cell cycle and cell proliferation (Figure S1A and S1B). Similar to TD cells, a mesenchymal-like cancer cell model with evident pro-metastatic potential, established via E-cadherin depletion, (shEcad) [19], also exhibited resistance to TGFβ-induced growth arrest (Figure S1C and S1D). Taken together, these results suggest that TD cells could serve as a model for the study of the ‘tumor-prone signaling shift’ that occurs upon TGFβ stimulation.

### Attenuated Smad-dependent TGFβ signaling caused by chronic TGFβ exposure

**Figure 2:**
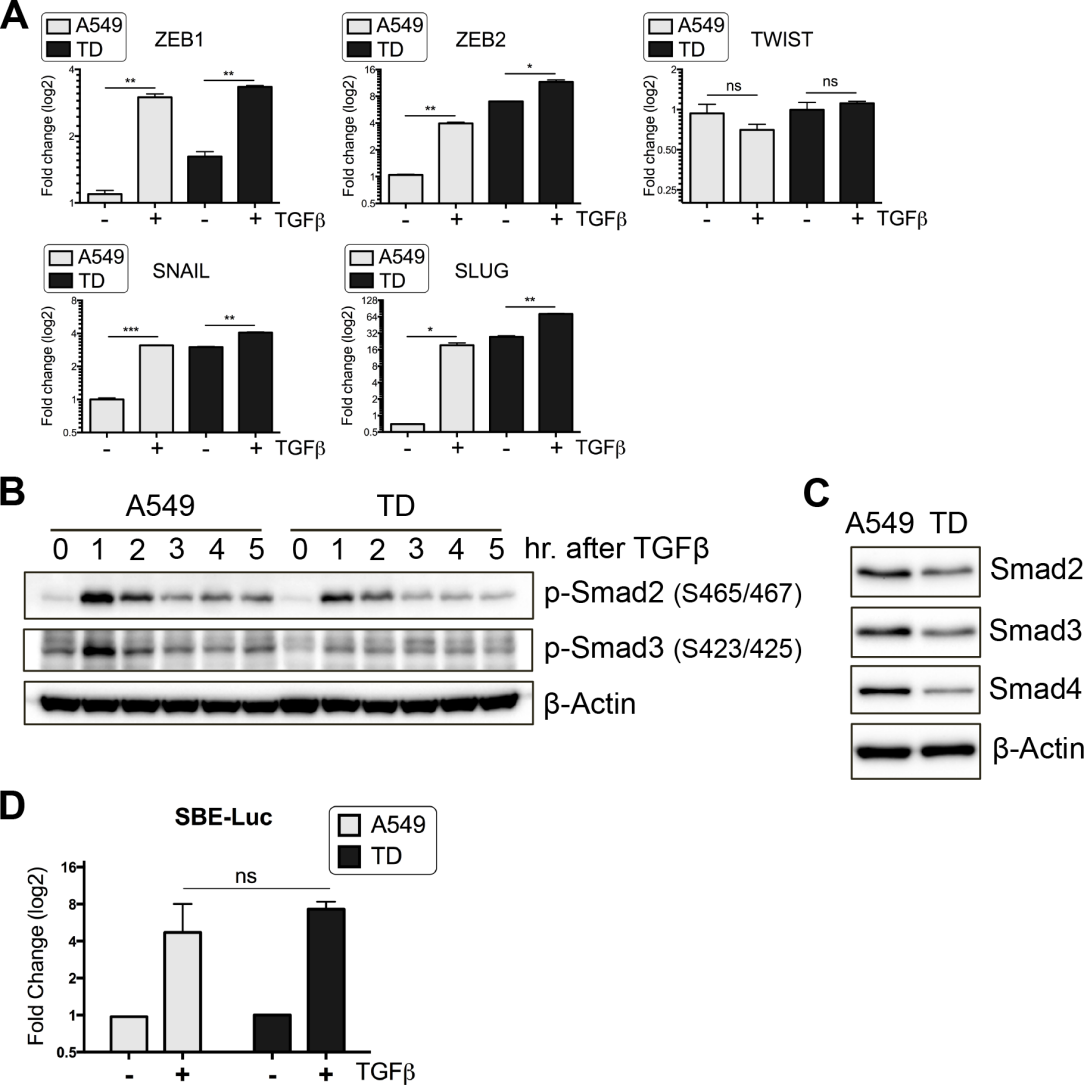
Attenuated Smad-dependent TGFβ signaling caused by chronic TGFβ exposure (**A**) After incubation in the presence or absence of TGFβ for 24 hours, transcript levels of TGFβ-inducible EMT genes were quantified with real-time PCR. (**B**) Immunoblotting was performed to determine time-dependent expression of phospho-Smad2 (Ser465/467) or phospho-Smad3 (Ser423/425) after TGFβ stimulation. (**C**) Total protein levels of Smad2, 3 and 4 were determined with immunoblotting. (**D**) The activity of TGFβ/Smad signaling was determined with SBE-Luciferase reporter systems. Luciferase activity was measured at 10 hour after TGFβ addition, and its fold induction is shown as graph. Columns, means ± SE. *P < 0.05; **P < 0.01; ***P < 0.001; ns, non-significant.

Considering the increased invasiveness of TGFβ-stimulated TD cells (Figure 1), we expected that TGFβ-dependent gene expression would be markedly induced in TD cells. However, unexpectedly, the relative fold change in TGFβ-induced mRNA expression of major EMT-regulatory factors (*ZEB1, ZEB2, TWIST, SNAIL* and *SLUG*), whose expression is positively regulated in a Smad-dependent manner [12], was markedly lower in TD cells than in control cells, despite the higher basal expression levels of these genes (except for *TWIST*) in TD cells (Figure 2A). More importantly, phosphorylation of Smad2 and Smad3 upon TGFβ stimulation was significantly attenuated in TD cells (Figure 2B), and the total protein expression level of not only Smad2 and Smad3 but also Smad4, a key co-factor for Smad2/3 nuclear translocation [20], was markedly reduced in TD cells (Figure 2C). To verify the Smad-dependent gene response, we employed Smad-binding element (SBE) luciferase (SBE-luc) reporter assays to determine TGFβ/Smad-dependent gene responses [21]. These assays revealed that TGFβ/Smad-dependent gene expression in TD cells was not more induced than in control cells (Figure 2D). Considering the above results, we decided to focus on non-canonical (Smad-independent) rather than canonical TGFβ pathways to elucidate the distinctive signaling features of TD cells.

### Increased Snail protein stability in TD cells

**Figure 3:**
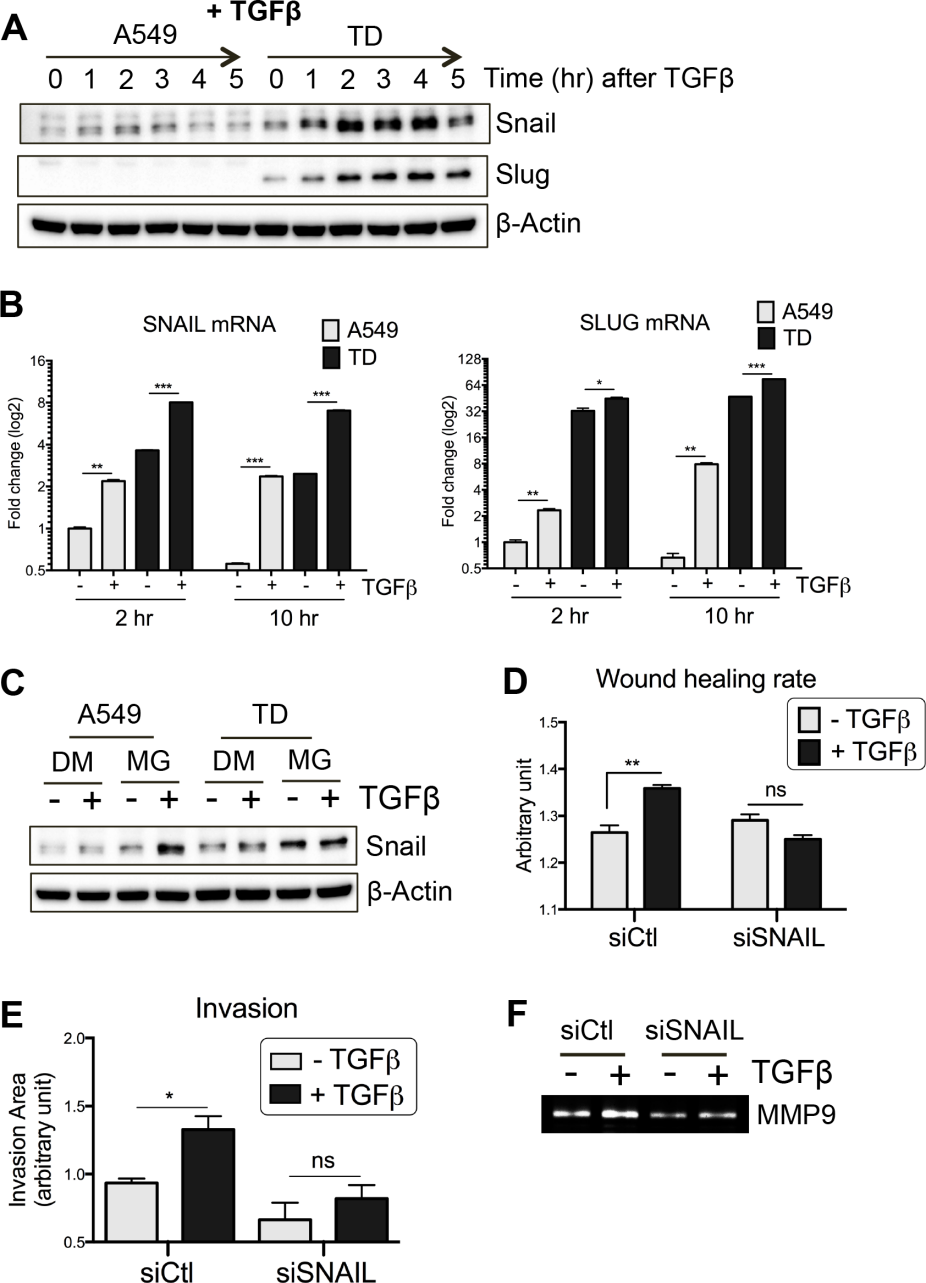
Increased Snail protein stability in TD cells (**A** and **B**) Time-dependent protein (A) and mRNA (B) induction of Snail and Slug after TGFβ stimulation was determined with immunoblotting and real-time PCR, respectively. (**C**) Stabilization of Snail protein was determined by MG132, a proteasome inhibitor, in the presence or absence of TGFβ. Cells were pretreated with MG132 (10 μM) for 30 minutes and subsequently stimulated with TGFβ for additional 1 hour. (**D**–**F**) The effect of Snail knockdown on TGFβ-induced invasiveness of TD cells was determined with wound healing assay (D), invasion assay (E) and zymography (F). TD cells were incubated with siRNA targeting SNAIL for 24 hours, followed by TGFβ stimulation and then subjected to wound healing and invasion assay. Supernatant of cell culture was subjected to zymography. DM, DMSO; MG, MG132. *Columns*, means ± SE. *P < 0.05; **P < 0.01; ***P < 0.001; ns, non-significant.

In addition to Smad-dependent transcriptional regulation, EMT-regulatory factors are also post-transcriptionally regulated by non-canonical TGFβ pathways [15, 16]. Thus, we examined the protein levels of key EMT factors in TD cells after TGFβ stimulation. Surprisingly, Snail and Slug protein levels were dramatically increased after TGFβ stimulation in TD cells within 2 hours (Figure 3A). Conversely, Zeb1 and Twist expression levels were not significant different between A549 and TD cells (data not shown). Considering the weak *SNAIL* and *SLUG* mRNA expression in TD cells after TGFβ exposure (at 2 hours and 10 hours) (Figure 3B), the increase in Snail and Slug protein levels in TD cells (at 2 hours) (Figure 3A) cannot be explained by Smad-dependent *SNAIL* or *SLUG* transcription alone (Figure 3B). Next, we searched the TCGA database (https://tcga-data.nci.nih.gov) to determine the significance of the *SNAIL* and *SLUG* mRNA expression levels in lung cancer patients (the Snail protein level is not available in the TCGA database). Both overall survival and post progression survival were negatively correlated with the expression level of *SNAIL*, while there was no reliable correlation in the case of *SLUG* (Figure S2A). These data suggest that Snail expression, but not Slug expression is clinically significant in lung cancer.

We next examined the possibility that the increased protein level of Snail in TD cells after TGFβ treatment results from increased Snail protein stabilization. The level of Snail was determined in the presence and absence of MG132, a proteasome inhibitor, after TGFβ treatment. In the presence of MG132, the amount of Snail in TD cells remained high regardless of TGFβ treatment, whereas the amount of Snail in control cells increased (Figure 3C). These results suggest that up-regulation of Snail protein stability rather than mRNA expression in TD cells is responsible for the induction of Snail at 2 hours after TGFβ treatment (Figure 3A).

To determine the effect of Snail induction after TGFβ treatment on TD cells, Snail was depleted using a knockdown approach. As shown in Figure S2B, Snail protein induction by TGFβ in TD cells was significantly decreased following knockdown, and the elevated invasion and migration capacity of TD cells was markedly suppressed (Figure 3D and 3E). Specifically, the enzymatic activity of MMP9, the expression of which strongly correlates with poor prognosis in lung cancer [22], was not activated by TGFβ treatment in Snail knockdown-TD cells (Figure 3F). Taken together, these data strongly suggest that, under chronic TGFβ stimulation, a high level of Snail protein expression is associated with increased metastatic potential of lung cancers.

### Inhibition of GSK3β by Akt is responsible for the increased stability of Snail

**Figure 4:**
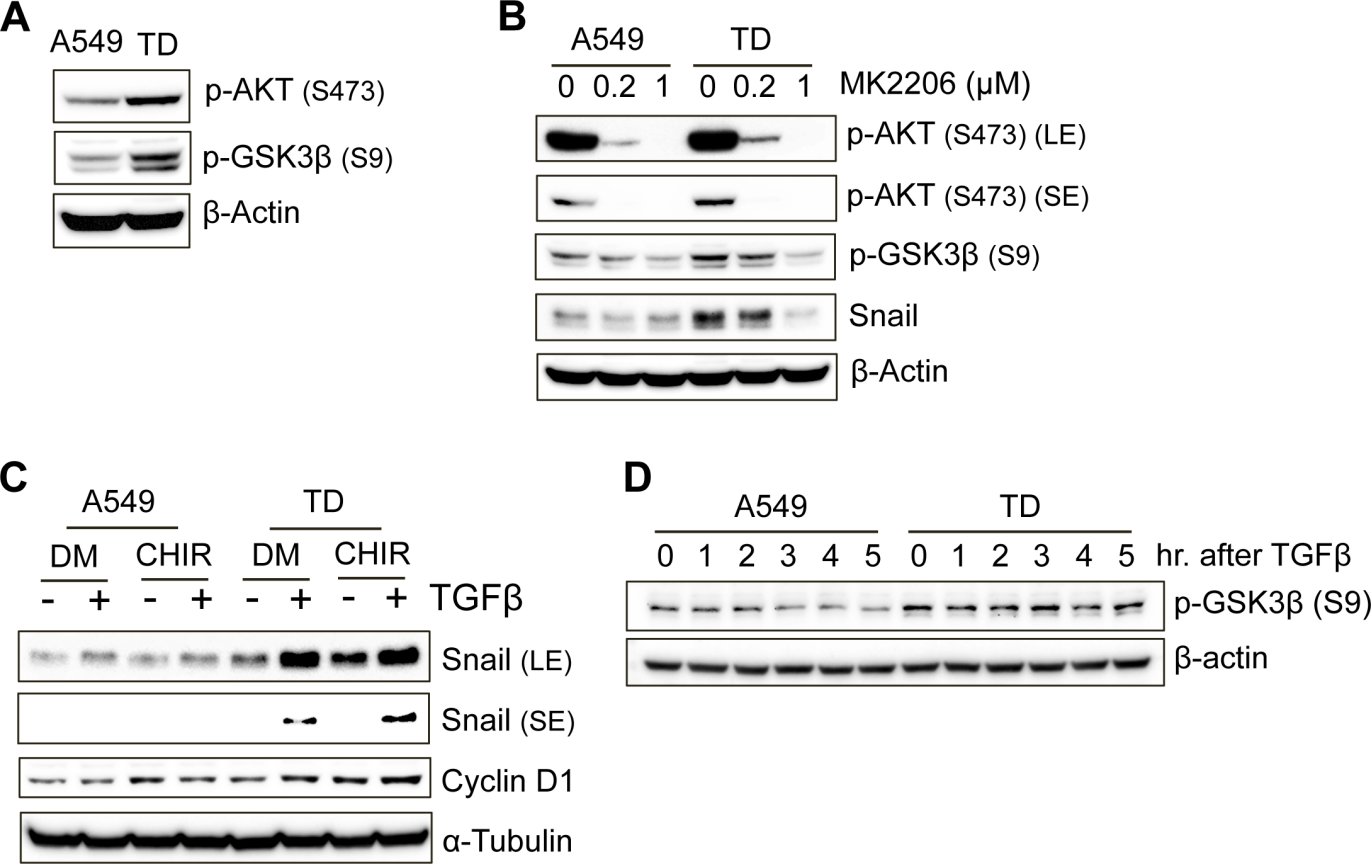
Inhibition of GSK3β by Akt is responsible for the increased stability of Snail (**A**) Immunoblotting for determining the phosphorylation levels of Akt (Ser473) and GSK3β (Ser9) in the basal culture condition. (**B**) The effect of MK2206, an Akt inhibitor, on GSK3β phosphorylation and Snail expression was determined with immunoblotting. MK2206 was treated at various concentrations for 3 hours before cell harvesting. (**C**) The inhibitory role of GSK3β on the stability of Snail protein was determined using a GSK3β-specific inhibitor, CHIR99021. Cells was pretreated with CHIR (3 μM) for 50 minutes and then incubated with or without TGFβ for 3 hour and 30 minutes before cell harvesting. The expression level of Snail protein was analyzed with immunoblotting. Cyclin D1, a substrate of GSK3β, was used as a positive control for the effect of CHIR. DM, DMSO; CHIR, CHIR99021. (**D**) Cells were harvested at the indicated time after TGFβ treatment, and then subjected to immunoblotting to determine the inhibitory phosphorylation levels of GSK3β.

We next examined whether the increased stability of Snail in TD cells after TGFβ treatment could have been caused by a decrease in the rate of its degradation. Because phosphorylation of Snail by GSK3β triggers ubiquitination of Snail and its degradation [16], determining GSK3β activity in TD cells is critical. As predicted, GSK3β phosphorylation, which leads to GSK3β inhibition and is governed by Akt activity, was high in TD cells. Consistently, Akt phosphorylation was higher in TD cells, an indication of active Akt, than in control cells (Figure 4A).

To prove that high Akt activity in TD cells was responsible for the high level of Snail protein, Snail protein levels were monitored following inhibition of Akt activity by treatment with MK2206, a potent Akt inhibitor currently used in clinical trials for the treatment of advanced solid tumors [23]. As shown in Figure 4B, Snail protein levels were markedly reduced while Akt active phosphorylation, as well as GSK3b inhibitory phosphorylation, was significantly reduced by Akt inhibitor treatment (Figure 4B). Conversely, GSK3β inhibition by CHIR99021 (CHIR) increased the level of Snail in TD cells. The extent of the increase was similar to that of the increase in the level of Cyclin D1, whose proteolysis is also determined by GSK3β-dependent phosphorylation [24] (Figure 4C). In the light of this, inhibitory phosphorylation of GSK3β remained high in TD cells after TGFβ stimulation, whereas it was slightly reduced in A549 cells (Figure 4D). These data indicate that the increase in the Snail protein level in TD cells (Figure 3A) is due to Snail protein stabilization mediated by increased Akt activity and subsequent inhibition of GSK3β activity.

### Increased integrin β3 expression in TD cells contributes to the activation of Akt-GSK3β-Snail signaling

**Figure 5:**
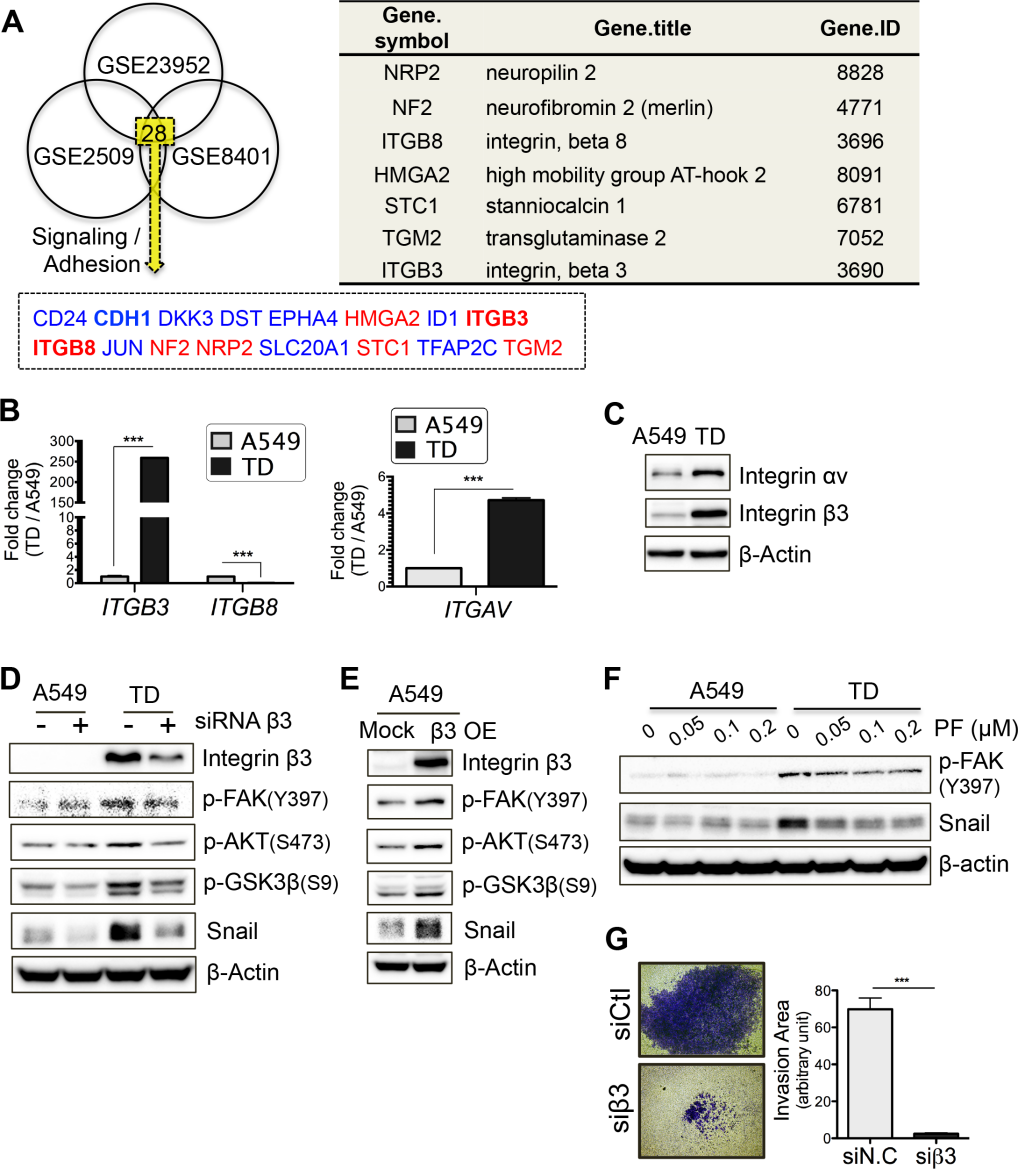
Increased integrin β3 expression in TD cells contributes to the activation of Akt-GSK3β-Snail signaling (**A**) GEO analysis to deduce candidate genes that regulate the distinctive signaling of TD cells. Three independent GSE studies including colon cancer cell (GSE2509), melanoma (GSE8401) and pancreatic cancer cell after TGFβ exposure (GSE23952) were used to select out common metastasis-regulating genes. Gene Ontology (GO) with ‘adhesion’ and ‘signaling’ was applied to narrow down candidate genes (left) and the gene information is shown in right table. Red and black indicate upregulated or downregulated, respectively. (**B** and **C**) Transcript levels of integrin β3, integrin β8 and integrin αv were analyzed with real-time PCR (B) and validated with immunoblotting against integrin αv or β3 (C). (**D** and **E**) Integrin β3 dependency toward the expression of its downstream signal components was evaluated with siRNA-mediated knockdown (D) and overexpression approach (E). siRNA against integrin β3 was introduced and after 2 day incubation, cell lysate was subjected to immunoblotting (D). And CMV expression vector encoding a human integrin β3 cDNA was transiently introduced and after 48 hours the cell lysate was subjected to immunoblotting (E). (**F**) FAK-dependent expression of Snail was determined. Cells were incubated with PF-562271, a FAK inhibitor, for 24 hours and immunoblotting was performed. (**G**) Contribution of integrin β3 on invasiveness of TD cells was analyzed. TD cells were introduced with siRNA Ctl (siCtl) or siRNA integrin β3 (siβ3) and were subjected to invasion assay. Representative images (left) and a bar graph (right), quantifying the invasiveness, are shown. *Columns*, means ± SE. *P < 0.05; **P < 0.01; ***P < 0.001; ns, non-significant.

The question remains as to how Akt activity was elevated in TD cells after TGFβ stimulation. Previously, we have reported that lung cancer cells become resistant to sheer stress after EMT [25], which is consistent with previously reported findings [26]. Based on this observation, we attempted to deduce a gene candidate(s) whose altered expression in TD cells contributes to both increased adhesion (e.g., resistance to sheer stress) and intracellular signaling (e.g., toward GSK3β). In this regard, we took advantage of GEO (http://www.ncbi.nlm.nih.gov/geo/) and chose three independent GSE studies that reported metastasis of colon (GSE2509), melanoma (GSE8401), and pancreatic cancer cells after TGFβ exposure (GSE23952) to find commonly altered gene(s) after metastasis (Figure S3A). We found 28 genes with altered expression (greater than two fold, *p* < 0.05) (Figure S3B). We further narrowed down these 28 candidates using Gene Ontology (GO) analysis with the terms “adhesion” and “signal”, and identified 16 gene candidates (nine downregulated genes and seven up-regulated genes) (Figure 5A). Of note, downregulation of CDH1 (encoding E-cadherin), an indicator of loss of epithelial features [27], was observed (Figure 5A), which validates this approach for identifying gene candidates. It seemed likely that one of the seven up-regulated genes (Figure 5A, right panel) contributed to ‘increased adhesion’ and ‘increased GSK3β phosphorylation’. Thus, we first focused on ITGB3 and ITGB8, which play critical role(s) in mediating adhesive signaling [28]. Moreover, there is emerging evidence that integrins are involved in cancer metastasis [29–31].

As predicted, ITGB3 but not ITGB8 expression was markedly increased in TD cells (Figure 5B and 5C). Additionally, integrin αv, a counterpart of integrin β3, was also increased in TD cells (Figure 5C), suggesting that the integrin αvβ3 complex might be involved in promoting the cancer-associated features of TD cells. It is worth noting that the integrin αvβ3 complex is also linked to cancer metastasis [32].

We next monitored phosphorylation of focal adhesion kinase (FAK), which is regulated by integrin engagement and which subsequently activates Akt [33], in the presence of knockdown or overexpression of integrin β3. As expected, phosphorylation of FAK (at Y397) and subsequent Akt activity were dependent on integrin β3 expression (Figure 5D and 5E). More importantly, Snail protein levels were also markedly reduced as a result of the depletion of integrin β3 in TD cells (Figure 5D) and were increased by ectopic expression of integrin β3 (β3 OE) in A549 control cells (Figure 5E). To further confirm the FAK-dependent regulation of Snail protein stabilization, the effect of PF562271, a FAK inhibitor, was evaluated. Consistently, the Snail protein level decreased in parallel with FAK inhibition by PF562271 (Figure 5F). Furthermore, the depletion of Snail (Figure 3E) or the loss of integrin β3 decreased the invasive capacity of the TD cells (Figure 5G). Taken together, these results indicate that a high expression level of integrin β3 contributes to the metastatic potential of TD cells via FAK-Akt-GSK3β axis-dependent stabilization of Snail.

### Prognostic significance of integrin β3 expression in lung cancer survival

**Figure 6:**
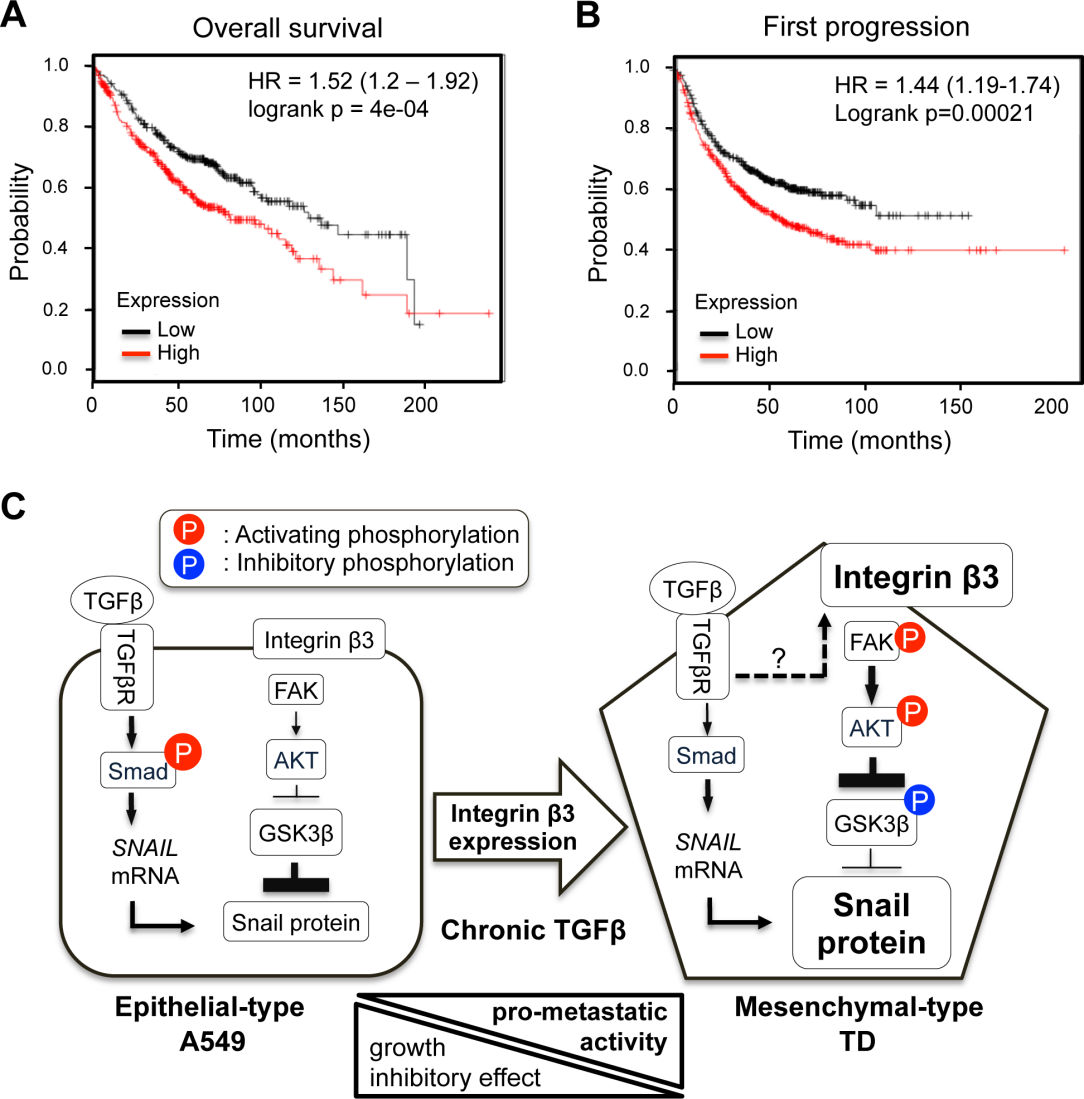
Prognostic significance of integrin β3 expression in lung cancer survival (**A** and **B**) Kaplan-Meier survival analysis and univariate Cox regression analysis were performed according to ITGB3 mRNA expression levels. (A) Overall survival, (B) First progression survival. The end point of first progression survival is the first relapse after surgery. (**C**) The proposed model explaining the signal signature of mesenchymal-type TD cells. Arrowline indicates ‘activation’, and perpendicular line indicates ‘inhibition’. Dashed arrowline indicates ‘upregulation’. P on a red background presents the activating phosphorylation, and P on a blue background presents the inhibitory phosphorylation.

To examine the prognostic significance of integrin β3 mRNA expression in lung cancer, survival analysis was carried out with a large clinical dataset (http://www.kmplot.com). The dataset was generated by combining several independent mRNA expression datasets from approximately 1, 700 lung cancer patients [34]. Survival analysis was performed on lung adenocarcinoma patients. The lung adenocarcinoma patients were divided into two subgroups, ITGB3-high and ITGB3-low, based on their median expression level of ITGB3. The KM curves showed a significant difference in the probability of both overall survival (*p* value = 4e-04) and first progression survival (*p* value = 0.0002) between the two groups (Figure 6). In univariate Cox regression analysis, the estimated hazard ratios for ITGB3-high, compared with ITGB3-low, were 1.52 and 1.44 for overall survival and first progression survival, respectively (Figure 6). Survival analysis showed that the expression levels of ITGB3 correlated with the prognosis of the lung adenocarcinoma patients, supporting the idea that high integrin β3 expression in TD cells resulting from chronic TGFβ exposure (e.g., mesenchymal cancer) is an important determinant of patient survival.

## DISCUSSION

It is widely accepted that cancer cells become refractory to TGFβ-mediated growth suppression and become more aggressive upon chronic exposure to TGFβ, once the cancer acquires mesenchymal properties [3]; this favors a TGFβ-enriched microenvironment. Therefore, identification of the molecular targets that govern the distinctive TGFβ signaling of mesenchymal cancer cells could help develop novel chemotherapeutic approaches to treating cancer.

To this end, we established a mesenchymal cancer model by exposing A549 tumor cells to chronic TGFβ exposure, which resulted in them becoming refractory to TGFβ-induced growth arrest and susceptible to TGFβ-induced tumor promotion. This model is similar to the irreversible mesenchymal cancer cell model induced by E-cadherin knockdown (Figure 1 and Figure S1). The ‘chronic TGFβ model (TD)’ is distinguishable from the ‘TGFβ-induced EMT model’ because the latter is achieved by a single treatment with TGFβ and its EMT phenotypes are transient [7]. Based on the roles of Snail in EMT and metastasis [37] and the poor prognostic outcomes of patients with high mRNA expression of Snail (Figure S2), we suggest that the increased Snail protein stability observed within 2 hours after treatment with TGFβ (Figure 3A), which increased the invasiveness of TD cells (Figure 3D and 3E), is important for the metastatic potential of mesenchymal cancer cells. Thus, the Akt activity toward GSK3β that leads to Snail protein stabilization could serve as a therapeutic target for reducing metastatic potential (Figure 4B). In agreement with this, increased Akt phosphorylation correlates positively with the poor prognosis of non-small cell lung cancer patients [38], emphasizing the clinical significance of high Akt activity in TD cells.

We further demonstrated that the increase in the integrin β3 level in several metastatic cancers with low E-cadherin expression, an indicator of loss of epithelial features [27] (Figure 5A), directed the adhesive signal toward the FAK and Akt signaling, which in turn affected Snail protein stability in TD cells (Figure 5). In this regard, Akt inhibitors, such as MK-2206 [23], or the FAK inhibitor, PF-562271 [39], could be used as starting points for the development of new drugs against mesenchymal cancer.

Identifying a pivotal signaling molecule that decides the metastatic capacity of a certain type of cancer would be highly advantageous for developing new drug targets. For example, induction of N-acetylgalactosaminyltransferase 14 (GalNAc-T14), whose expression is extremely low in normal lung tissue [40], in specific types of cancer cells contributes to metastasis through Wnt signaling [41]. Thus, a β-catenin inhibitor that abrogates the Wnt-dependent gene responses is highly effective in inhibiting metastasis in GalNAc-T14-expressing cancers [41]. Similarly, because its high expression indicates a poor prognostic outcome (Figure 6) and tyrosine kinase inhibitor (TKI) resistance [42], integrin β3 could serve as an indicator for determining whether or not a therapeutic regimen consisting of Akt or FAK inhibitors would be suitable for the treatment of mesenchymal cancers expressing high levels of Snail protein, as summarized in Figure 6D [42].

In summary, chronic TGFβ promotes the metastatic potential of lung adenocarcinoma by increasing Snail protein stability via its effect on the integrin β3-FAK-Akt-GSK3β signaling axis, suggesting that integrin β3 could be used as an indicator for determining the effectiveness of Akt- or FAK-targeted therapies.

## MATERIALS AND METHODS

### Establishment of TD cells and the use of TGFβ1

To generate transdifferentiated A549 (TD) cells, A549 cells were repeatedly treated with TGFβ1 (CYT-716, PROSPEC) at 2 ng/ml concentration every 3 days for 60 days and then TD cells were divided and frozen into a number of vials. For further experiments, newly thawed frozen TD cells were used for 30 days, during which TD cells were also exposed to repetitive TGFβ at 2 ng/ml concentration every 3 days, thus 60~90 day TGFβ1-exposed TD cells being used in this study. And TGFβ1 was used at 5 ng/ml concentration in all experiments with additional TGFβ1 treatment.

### Invasion and migration assay

For invasion assay, trans-wells (6.5 mm) with 8 μm pore polycarbonate membrane inserts (Corning) were embedded with matrigel (BD) in DMEM. Cells (1 × 10^5^ per well) were added into the Matrigel-embedded inserts (the top chambers) and the inserts were placed into the bottom chambers containing DMEM media. After 24 hours, the membranes were fixed with 4% formaldehyde in PBS for 10 min, and the invading cells on the membranes were stained with 0.1% crystal violet. Wound healing assay was performed in low serum (0.2% FBS) condition. A wound was formed by scratching a confluent cell monolayer and after that the images were taken. Migration area was calculated by subtracting the scratched area from the total area of image.

### Zymography

The supernatants of cell culture were concentrated using centricon (Millipore, 30 kDa cut), and added with non-reducing sample buffer (without DTT). The samples were loaded into the SDS-PAGE gel-containing gelatin B (Sigma-Aldrich). After electrophoresis, gel was washed with 2.5% Triton X-100 in Tris-HCL buffer (pH 7.5). Then the gel was incubated with reaction buffer (15 mM Nacl, 10 mM CaCl_2_ in Tris-HCl butter (pH 7.5)) overnight at room temperature to induce gelatin lysis by MMP9, and then stained with Coomassie brilliant blue for 30 min followed by destaining.

## SUPPLEMENTARY MATERIALS FIGURES



## References

[1] Bachman KE, Park BH (2005). Duel nature of TGF-beta signaling: tumor suppressor vs. tumor promoter. Current opinion in oncology.

[2] Padua D, Massague J (2009). Roles of TGFbeta in metastasis. Cell Res.

[3] Roberts AB, Wakefield LM (2003). The two faces of transforming growth factor beta in carcinogenesis. Proc Natl Acad Sci U S A.

[4] Pardali K, Moustakas A (2007). Actions of TGF-beta as tumor suppressor and pro-metastatic factor in human cancer. Biochim Biophys Acta.

[5] Derynck R, Akhurst RJ, Balmain A (2001). TGF-beta signaling in tumor suppression and cancer progression. Nature genetics.

[6] Massague J (2008). TGFbeta in Cancer. Cell.

[7] Gal A, Sjoblom T, Fedorova L, Imreh S, Beug H, Moustakas A (2008). Sustained TGF beta exposure suppresses Smad and non-Smad signalling in mammary epithelial cells, leading to EMT and inhibition of growth arrest and apoptosis. Oncogene.

[8] Ito D, Fujimoto K, Doi R, Koizumi M, Toyoda E, Mori T, Kami K, Kawaguchi Y, Whitehead R, Imamura M (2004). Chronic exposure of transforming growth factor beta 1 confers a more aggressive tumor phenotype through downregulation of p21(WAF1/CIP1) in conditionally immortalized pancreatic epithelial cells. Surgery.

[9] Sheng H, Shao J, O'Mahony CA, Lamps L, Albo D, Isakson PC, Berger DH, DuBois RN, Beauchamp RD (1999). Transformation of intestinal epithelial cells by chronic TGF-beta1 treatment results in downregulation of the type II TGF-beta receptor and induction of cyclooxygenase-2. Oncogene.

[10] Bierie B, Moses HL (2006). Tumour microenvironment: TGFbeta: the molecular Jekyll and Hyde of cancer. Nat Rev Cancer.

[11] Wang LH, Kim SH, Lee JH, Choi YL, Kim YC, Park TS, Hong YC, Wu CF, Shin YK (2007). Inactivation of SMAD4 tumor suppressor gene during gastric carcinoma progression. Clin Cancer Res.

[12] Heldin CH, Vanlandewijck M, Moustakas A (2012). Regulation of EMT by TGFbeta in cancer. FEBS Lett.

[13] Zhang YE (2009). Non-Smad pathways in TGF-beta signaling. Cell Res.

[14] Weiss MB, Abel EV, Mayberry MM, Basile KJ, Berger AC, Aplin AE (2012). TWIST1 is an ERK1/2 effector that promotes invasion and regulates MMP-1 expression in human melanoma cells. Cancer Res.

[15] Vichalkovski A, Gresko E, Hess D, Restuccia DF, Hemmings BA (2010). PKB/AKT phosphorylation of the transcription factor Twist-1 at Ser42 inhibits p53 activity in response to DNA damage. Oncogene.

[16] Zhou BP, Deng J, Xia W, Xu J, Li YM, Gunduz M, Hung MC (2004). Dual regulation of Snail by GSK-3beta-mediated phosphorylation in control of epithelial-mesenchymal transition. Nat Cell Biol.

[17] Zhang B, Zhang B, Chen X, Bae S, Singh K, Washington MK, Datta PK (2014). Loss of Smad4 in colorectal cancer induces resistance to 5-fluorouracil through activating Akt pathway. Br J Cancer.

[18] Zhang L, Zhou F, ten Dijke P (2013). Signaling interplay between transforming growth factor-beta receptor and PI3K/AKT pathways in cancer. Trends Biochem Sci.

[19] Bae GY, Choi SJ, Lee JS, Jo J, Lee J, Kim J, Cha HJ (2013). Loss of E-cadherin activates EGFR-MEK/ERK signaling, which promotes invasion via the ZEB1/MMP2 axis in non-small cell lung cancer. Oncotarget.

[20] Massague J, Seoane J, Wotton D (2005). Smad transcription factors. Genes Dev.

[21] Moustakas A, Souchelnytskyi S, Heldin CH (2001). Smad regulation in TGF-beta signal transduction. Journal of cell science.

[22] Cox G, Jones JL, O'Byrne KJ (2000). Matrix metalloproteinase 9 and the epidermal growth factor signal pathway in operable non-small cell lung cancer. Clin Cancer Res.

[23] Yap TA, Yan L, Patnaik A, Fearen I, Olmos D, Papadopoulos K, Baird RD, Delgado L, Taylor A, Lupinacci L, Riisnaes R, Pope LL, Heaton SP (2011). First-in-man clinical trial of the oral pan-AKT inhibitor MK-2206 in patients with advanced solid tumors. Journal of clinical oncology.

[24] Diehl JA, Cheng M, Roussel MF, Sherr CJ (1998). Glycogen synthase kinase-3beta regulates cyclin D1 proteolysis and subcellular localization. Genes Dev.

[25] Kim M-J, Doh I, Bae G-Y, Cha H-J, Cho Y-H (2014). Cell-matrix adhesion characterization using multiple shear stress zones in single stepwise microchannel. Applied Physics Letters.

[26] Voulgari A, Pintzas A (2009). Epithelial-mesenchymal transition in cancer metastasis: mechanisms, markers and strategies to overcome drug resistance in the clinic. Biochim Biophys Acta.

[27] Tsai JH, Yang J (2013). Epithelial-mesenchymal plasticity in carcinoma metastasis. Genes Dev.

[28] Hynes RO (1992). Integrins: versatility, modulation, and signaling in cell adhesion. Cell.

[29] Ganguly KK, Pal S, Moulik S, Chatterjee A (2013). Integrins and metastasis. Cell Adh Migr.

[30] Bendas G, Borsig L (2012). Cancer cell adhesion and metastasis: selectins, integrins, and the inhibitory potential of heparins. International journal of cell biology.

[31] Hart IR (2004). Role of integrins in tumor invasion and metastasis. Exp Dermatol.

[32] Bacac M, Stamenkovic I (2008). Metastatic cancer cell. Annu Rev Pathol.

[33] Guo W, Giancotti FG (2004). Integrin signalling during tumour progression. Nat Rev Mol Cell Biol.

[34] Gyorffy B, Surowiak P, Budczies J, Lanczky A (2013). Online survival analysis software to assess the prognostic value of biomarkers using transcriptomic data in non-small-cell lung cancer. PLoS One.

[35] Tian M, Neil JR, Schiemann WP (2011). Transforming growth factor-beta and the hallmarks of cancer. Cell Signal.

[36] Hanahan D, Weinberg RA (2011). Hallmarks of cancer: the next generation. Cell.

[37] Barrallo-Gimeno A, Nieto MA (2005). The Snail genes as inducers of cell movement and survival: implications in development and cancer. Development.

[38] Tang JM, He QY, Guo RX, Chang XJ (2006). Phosphorylated Akt overexpression and loss of PTEN expression in non-small cell lung cancer confers poor prognosis. Lung Cancer.

[39] Golubovskaya VM (2014). Targeting FAK in human cancer: from finding to first clinical trials. Frontiers in bioscience.

[40] Stern HM, Padilla M, Wagner K, Amler L, Ashkenazi A (2010). Development of immunohistochemistry assays to assess GALNT14 and FUT3/6 in clinical trials of dulanermin and drozitumab. Clin Cancer Res.

[41] Kwon OS, Oh E, Park JR, Lee JS, Bae GY, Koo JH, Kim H, Choi YL, Choi YS, Kim J, Cha HJ (2015). GalNAc-T14 promotes metastasis through Wnt dependent HOXB9 expression in lung adenocarcinoma. Oncotarget.

[42] Seguin L, Desgrosellier JS, Weis SM, Cheresh DA (2015). Integrins and cancer. Trends Cell Biol.

