# Supplementary material for: Chronic TGFβ stimulation promotes the metastatic potential of lung cancer cells by Snail protein stabilization through integrin β3-Akt-GSK3β signaling
Source: Oncotarget. 2016 Mar 23;7(18):25366–76. doi: 10.18632/oncotarget.8295 (PMC5041910; doi:10.18632/oncotarget.8295)
Supplement: Supplementary file 1 [file oncotarget-07-25366-s001.pdf]

Chronic TGFβ stimulation promotes the metastatic potential of lung cancer cells by Snail protein stabilization through integrin β3-Akt-GSK3β signaling

Supplementary Materials

SUPPLEMENTARY MATERIALS AND METHODS

Real-time PCR

Total cellular RNA was extracted using Trizol, followed by RT-PCR to generate the first strand cDNA, and the cDNA was subjected to SYBR Green-based Real-time PCR (Roche LightCycler 480 II).

Immunofluorescence cytochemistry (IFC)

Cells were plated on glass coverslips and allowed to grow for overnight. For E-cadherin staining, cells were washed in PBS, fixed with 4% paraformaldehyde for 10 min at room temperature, and then permeabilized with 0.1% Triton X-100 for 2 min. After blocking with 3% BSA for 1 hr, cells were incubated with diluted (1:200) E-cadherin antibody for 1 hr. Cells were washed in PBS followed by addition of secondary antibody conjugated to a flurochrome (Jackson Laboratory), phalloidin and DAPI in blocking solution for 1 hr. Cells were washed in PBS and cover-slipped, and examined with a fluorescent microscope (Olympus, BX 53).

Statistical analysis

The graphical data were presented as mean ± S.E.M. Statistical significance among the three groups and between groups was determined using one-way or two-way analysis of variance (ANOVA) following Bonferroni post-test and Student's *t*-test respectively. Significance was assumed for $p < 0.05$ (*), $p < 0.01$ (**), $p < 0.001$ (***).

Reagents and antibodies

siRNAs targeting *SNAIL* (cat# 1141904) and *ITGB3* (cat# 1075876) respectively were obtained from Bioneer and CMV mammalian expression vector encoding a human integrin β3 cDNA and SBE-Luc reporter plasmid were obtained from Addgene. MK2206 (cat# S1078), PF562271 (cat# S2672) and CHIR99021 (cat# S2924) were obtained from SelleckChem. Antibodies against phospho-Smad2 (S465/467) (#3101), phospho-Smad3 (S423/425) (#9520), phospho-Akt (S473) (#4060), phospho-GSK3β (S9) (#9336), phospho-FAK (Y397) (#8556), Smad2 (#3103), Smad3 (#9523), Snail (#3879), Slug (#9585), Integrin β3 (#4702), E-cadherin (#4065) and N-cadherin (#4061) were obtained from Cell Signaling Technology. Antibodies against β-Actin (sc-47778), Smad4 (sc-7966), CyclinD1 (sc-718) and α-Tubulin (sc-8035) were obtained from Santa Cruz Biotechnology Inc. Integrin αv (A2091) antibody was obtained from Neobiolab.

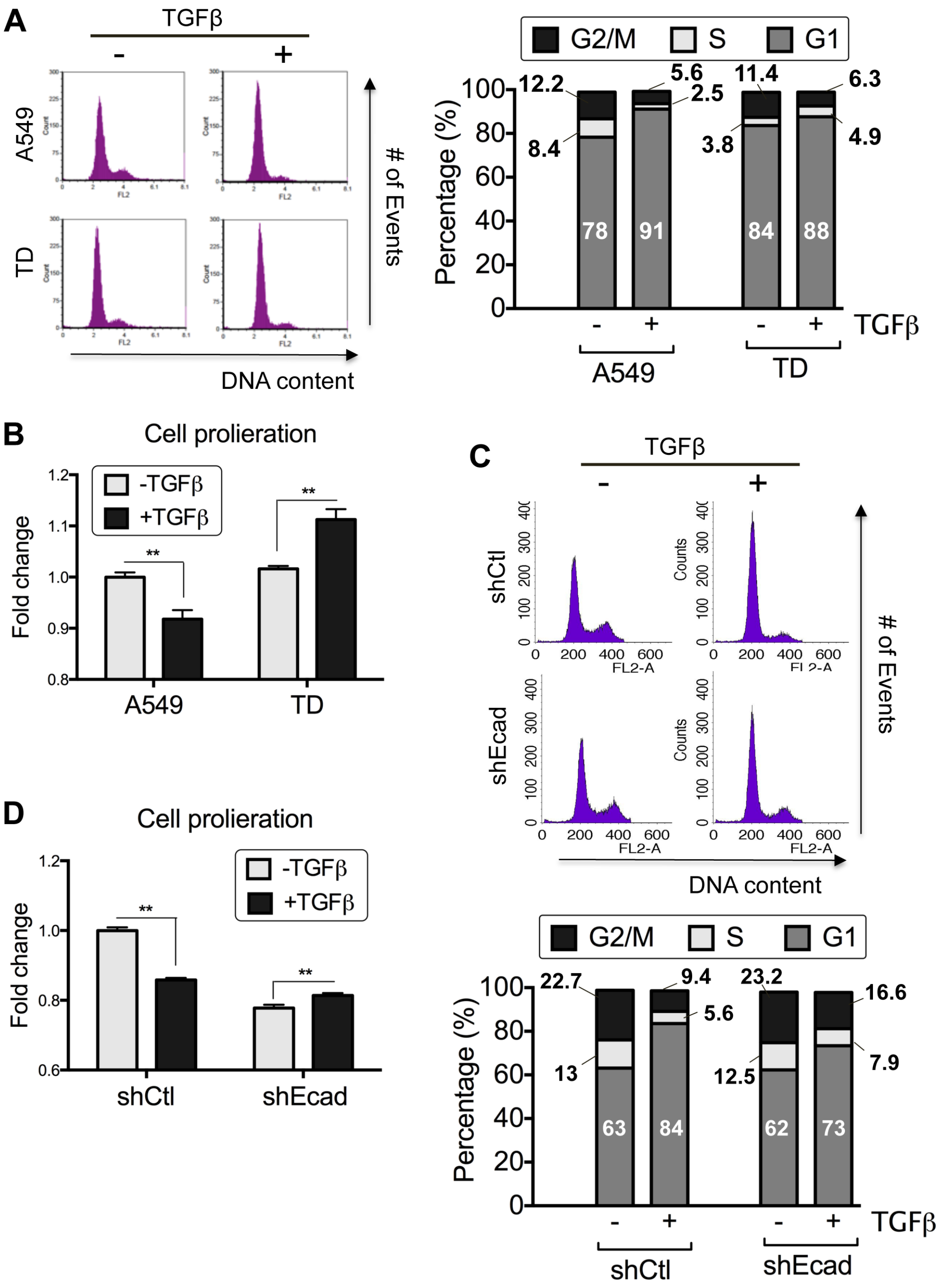

Supplementary Figure S1: Resistance to the growth inhibitory effect of TGFβ was acquired by chronic TGFβ exposure or loss of E-cadherin. (**A** and **C**) Cells; A549 vs. TD (A) and shCtl vs. shEcad (C), incubated with or without TGFβ for 24 hours, were subjected to P.I staining and FACS analysis. Naive images, presenting the population for each cell cycle and calculated values (as a graph) are shown. (**B** and **D**) After TGFβ treatment for 24 hours, cell proliferation was determined with XTT assay. A549 vs. TD (B) and shCtl vs. shEcad (D).

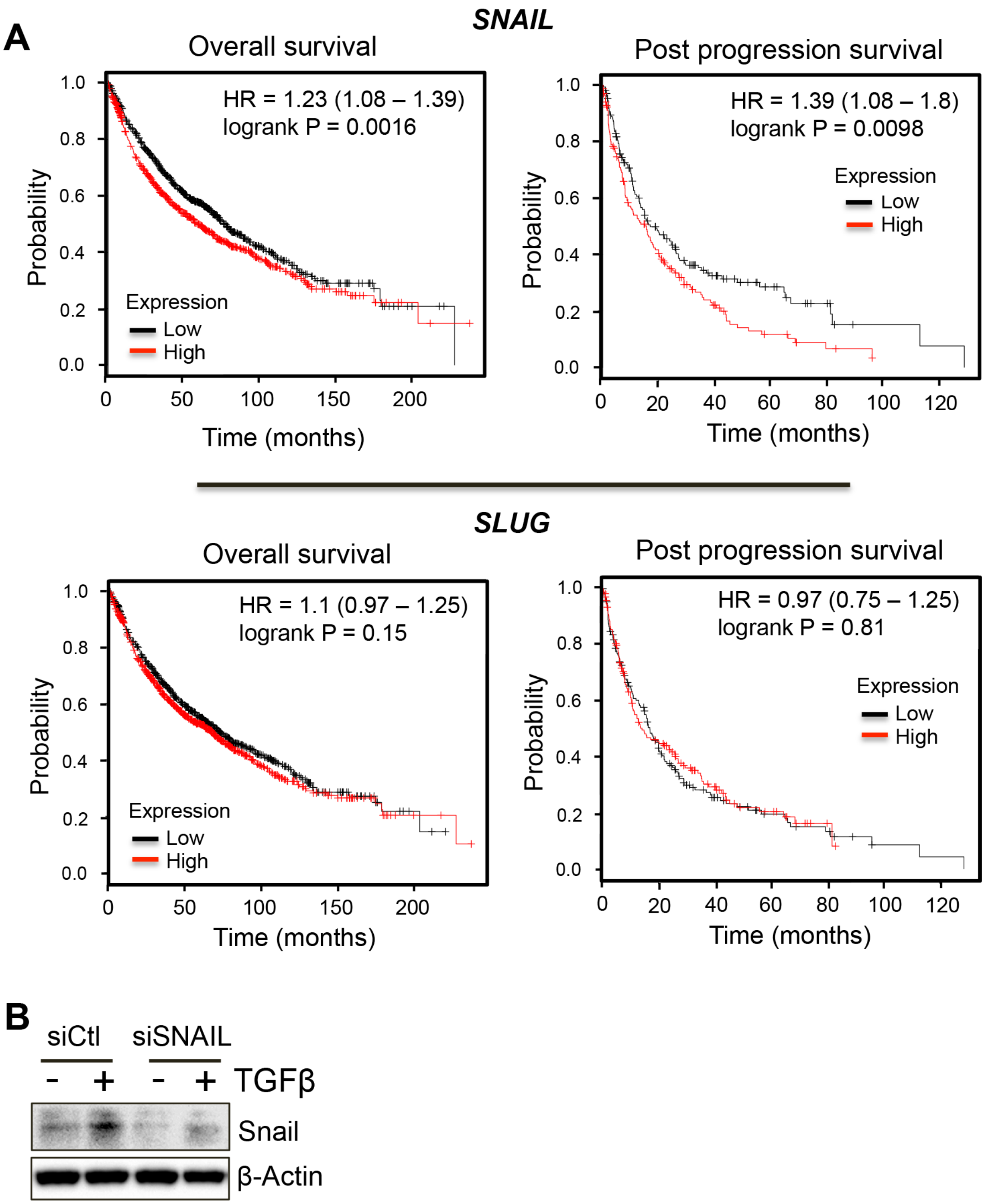

Supplementary Figure S2: Prognostic significance of *SNAIL* expression in lung cancer patients. (**A**) Overall survival or post progression survival curve for *SNAIL* or *SLUG* mRNA expression were obtained from KM plotter for lung cancers (http://www.kmplot.com). (**B**) siRNA-mediated *SNAIL* knockdown was validated with immunoblotting.

A

GSE number	Cancer model	Cell line	Condition	Sample size	Reference (PMID#)
GSE23952	Pancreatic	Panc-1	TGFβ exposure	Control (3), TGFβ (3)	20885998
GSE2509	Colorectal	SW480 SW620	Lymph node metastasis (SW620) *vs.* primary tumor (SW480)	SW480 (3), SW620 (3)	16531451
GSE8401	Melanoma	Met Nr Prim Nr	Metastases *vs.* Primary tumors	Primary tumors (31), Metastases (52)	18505921

B

	Gene. symbol	Gene.title	Gene.ID
up-regulated	**NRP2**	neuropilin 2	8828
	**NF2**	neurofibromin 2 (merlin)	4771
	**ITGB8**	integrin, beta 8	3696
	**HMGA2**	high mobility group AT-hook 2	8091
	**STC1**	stanniocalcin 1	6781
	**ITGB3**	integrin, beta 3 (platelet glycoprotein IIIa, antigen CD61)	3690
	**TGM2**	transglutaminase 2	7052
Down-regulated	**AQP3**	aquaporin 3 (Gill blood group)	360
	**CD24**	CD24 molecule	100133941
	**CDH1**	cadherin 1, type 1, E-cadherin (epithelial)	999
	**CLTB**	clathrin, light chain B	1212
	**CTSV**	cathepsin V	1515
	**DKK3**	dickkopf WNT signaling pathway inhibitor 3	27122
	**DST**	dystonin	667
	**EPHA4**	EPH receptor A4	2043
	**HPGD**	hydroxyprostaglandin dehydrogenase 15-(NAD)	3248
	**ID1**	inhibitor of DNA binding 1, dominant negative helix-loop-helix protein	3397
	**JUN**	jun proto-oncogene	3725
	**LAMC2**	laminin, gamma 2	3918
	**MCAM**	melanoma cell adhesion molecule	4162
	**MYL9**	myosin, light chain 9, regulatory	10398
	**MYO1D**	myosin ID	4642
	**NEBL**	nebulette	10529
	**QPCT**	glutaminyl-peptide cyclotransferase	25797
	**RAB27B**	RAB27B, member RAS oncogene family	5874
	**S100A2**	S100 calcium binding protein A2	6273
	**SLC20A1**	solute carrier family 20 (phosphate transporter), member 1	6574
	**TFAP2C**	transcription factor AP-2 gamma (activating enhancer binding protein 2 gamma)	7022

Supplementary Figure S3: GEO analysis to deduce commonly regulated genes in three independent GSE studies. (**A**) The information about GSE studies used in this study. (**B**) Full name and ID about selected genes are shown. Red and blue indicate upregulated or downregulated, respectively.

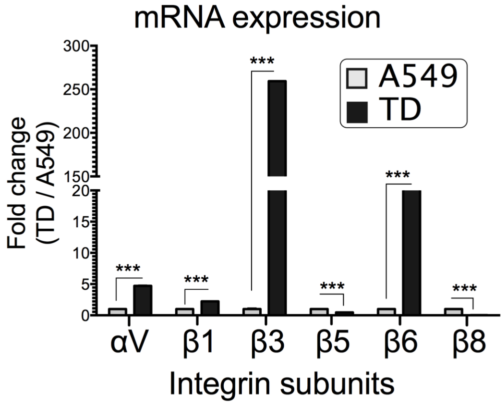

Supplementary Figure S4: In the basal culture condition, A549 or TD cells were harvested 2 days after split to analyze mRNA levels of integrin αV-family integrins with quantitative real-time PCR.
